# Early life oxytocin treatment improves thermo-sensory reactivity and maternal behavior in neonates lacking the autism-associated gene *Magel2*

**DOI:** 10.1038/s41386-022-01313-5

**Published:** 2022-04-08

**Authors:** Laura Caccialupi Da Prato, Ugo Zayan, Dina Abdallah, Vanessa Point, Fabienne Schaller, Emilie Pallesi-Pocachard, Aurélie Montheil, Stéphane Canaan, Jean-Luc Gaiarsa, Françoise Muscatelli, Valéry Matarazzo

**Affiliations:** 1 Aix Marseille Univ, INSERM, INMED, Marseille, France; 2 Aix-Marseille Univ, CNRS, LISM, IMM, Marseille, France

**Keywords:** Autism spectrum disorders, Perception

## Abstract

Atypical responses to sensory stimuli are considered as a core aspect and early life marker of autism spectrum disorders (ASD). Although recent findings performed in mouse ASD genetic models report sensory deficits, these were explored exclusively during juvenile or adult period. Whether sensory dysfunctions might be present at the early life stage and rescued by therapeutic strategy are fairly uninvestigated. Here we found that under cool environment neonatal mice lacking the autism-associated gene *Magel2* present pup calls hypo-reactivity and are retrieved with delay by their wild-type dam. This neonatal atypical sensory reactivity to cool stimuli was not associated with autonomic thermoregulatory alteration but with a deficit of the oxytocinergic system. Indeed, we show in control neonates that pharmacogenetic inactivation of hypothalamic oxytocin neurons mimicked atypical thermosensory reactivity found in *Magel2* mutants. Furthermore, pharmacological intranasal administration of oxytocin to *Magel2* neonates was able to rescue both the atypical thermosensory response and the maternal pup retrieval. This preclinical study establishes for the first-time early life impairments in thermosensory integration and suggest a therapeutic potential benefit of intranasal oxytocin treatment on neonatal atypical sensory reactivity for autism.

## Introduction

Autism spectrum disorder (ASD) is a developmental disorder characterized by challenges with social interaction, speech and non-verbal communication, as well as repetitive behaviors. However, atypical sensory behaviors are a core aspect of ASD affecting 90% of children [1]. Importantly, atypical sensory sensitivities have been documented as early as 6 months in ASD infants, preceding considerably the common core features and the diagnosis. Recent increasing evidences suggest that sensory traits such as tactile, visual, auditory, olfactory, gustatory and heat abnormalities are present in juvenile and adult ASD models [2–9]. However, investigation of sensory dysfunctions during early post-natal development of ASD models is an important step in early diagnosis and development of therapeutic strategies for ASD.

During the first week of life, sensory integrity is instrumental since neonates have to undertake vital innate behaviors such as nipple-searching and alert calls. Among the various stimuli arising from the external world, sensing any reduction of the ambient temperature is particularly relevant for neonatal pups. Indeed, unlike their homeothermic adult counterparts, neonates are poikilothermic [10] and should be kept in close contact with the mother or their littermates in order to keep their body temperature. In the absence of their warmth-giving mother and being exposed to cool temperatures, neonate mice generate ultrasonic vocalizations (USV). In fact, during the perinatal period, exposure to low ambient temperatures is considered as a major stimulus eliciting USV [11–13]. Interestingly, atypical early vocalization has been detected in 6-month-old infants at risk for autism [14] and may represent an early biomarker for ASD [15].

The neuronal pathways underlying cool response behavior are still under intensive investigation. At the peripheral level, thermosensory neurons have been described in the skin and in the Grueneberg ganglion (GG) – a cluster of sensory neurons localized at the tip of the nose [16–19]. It has been proposed that this ganglion influences USV [17, 20] generated by rodent neonates to elicit maternal care on exposure to cool temperatures [11, 13, 21].

Following cool exposure, newborns require an effective thermoregulatory adaptive response to produce heat [22]. During this period of development, they are unable to shiver and the primary source of heat is produced by the sympathetically mediated metabolism of brown adipose tissue (BAT); named non-shivering thermogenesis [23].

Here, using behavioral tests to assess neonatal thermosensory reactivity, we discovered the existence of early developmental deficits in thermal sensitivity in neonate mice lacking the autism-associated gene *Magel2*. *MAGEL2* is an imprinted gene highly expressed in the hypothalamus that is paternally expressed and which paternal deletion and point mutation cause Prader-Willi [24] and Schaaf-Yang [25] respectively; two syndromes with high prevalence of ASD (27% and 78% respectively). The patients have sensory disorders characterized by instability of body temperature manifested with episodes of hyper or hypothermia without infectious causes and which can be fatal in infants [26, 27]. Moreover, adolescent with ASD present a decreased sensitivity to thermal stimuli [28].

With the aim to explore the physio-pathophysiological mechanism underlying this thermal deficit we explored peripheral functional activities of both GG and BAT. Furthermore, since the oxytocinergic system is considered as a rheostat of adult sensory functions [29], a modulator of huddling and thermotaxis behavior in response to cold challenge [30] and it is altered in *Magel2*^*+/−p*^ neonate mice [31, 32], we investigated whether central dysfunction of the oxytocinergic system could sustain neonatal thermosensory reactivities and whether neonatal oxytocin (OT) pharmacological treatment could be a therapeutic approach.

Finally, whilst OT is indeed important for maternal care behavior [33], in this study the manipulation of temperature and OT occurs only in the pup, there were no modification of OT in the dam which are all WT.

## Material And methods

### Animals

Mice were handled and cared in accordance with the Guide for the Care and Use of Laboratory Animals (N.R.C., 1996) and the European Communities Council Directive of September 22nd, 2010 (2010/63/EU, 74). Experimental protocols were approved by the institutional Ethical Committee Guidelines for animal research with the accreditation no. B13–055–19 from the French Ministry of Agriculture. All efforts were made to minimize the number of animals used. 129-Gt(ROSA)26Sor^tm1(CAG-CHRM4*,-mCitrine)Ute^ also known as R26-LSL-hM4Di DREADD were obtained from the Jackson Laboratory (stock #026219) and called here hM4Di DREADD mice for convenience. Due to the parental imprinting of *Magel2* only heterozygous pups (+m/-p) with the mutated allele transferred from the male were used for experiments. The OT-cre mice were obtained from the Jackson Laboratory (stock #24234). In our experiment we used hM4Di DREADD homozygous // heterozygous OT-cre mice (referred here as OT hM4DI).

### Neonatal thermoregulatory behavior and USV recording

On the day of testing (P0, P1, P2, P3 and P6), after 30 min of room habituation in the testing room, each pup was separated from its littermates and dam, placed on a heating pad and each pup were isolated in a box (23 × 28 × 18 cm) located inside an anechoic box (54 × 57 × 41 cm; Couldbourn instruments, PA, USA) for a 5 min test at room temperature (25 °C). Then the pup goes back to the dam for 5–10 min and is submitted to a second separation, placed on a heating pad and the USV were recorded during 5 min under cool temperature (17 °C). We conducted reversal assays (17 °C then 25 °C) to exclude any potentiation effect. An ultrasound microphone (Avisoft UltraSoundGate condenser microphone capsule CM16/CMPA, Avisoft bioacoustics, Germany) sensitive to frequencies of 10–250 kHz was located in the roof of the isolation box. Recordings were done using Avisoft recorder software (version 4.2) with a sampling rate of 250 kHz in 16 bits format. Data were transferred to SASLab Pro software (version 5.2; Avisoft bioacoustics) and a fast Fourier transformation was conducted (256 FFT-length, 100% frame, Hamming window, and 75%-time window overlap) before the analysis. Recordings were analyzed for the number of calls during the 5 min recording at 25 °C and 17 °C and for the latency which is the first ultrasound call of the record. The cooling responsive rate was calculated as the proportion of pups responsive to cooling: a pup is responsive if the latency is twice as short at 17 °C than at 25 °C.

### Maternal pup retrieval behavior

Both WT and *Magel2* pups (P2) were born and raised by a WT dam. Female (Dam or virgin female) were placed in a behavioral arena (40 × 30 × 15 cm) containing two zones: the nest zone with the nesting material and the pup’s zone, both separated by a wall with a door allowing the female to cross from one zone to the other. Female were given 30 minutes to acclimate before each testing session began. Pups preserved on a heating pad. Each session of testing consisted of series of trials during which each pup of the litter was put one after the other in the pup’s zone in a given temperature (25 °C then 17 °C). Before and after testing all pups of the littermate, one object was inserted into the pup’s zone for control. The female was given 3 minutes to retrieve the displaced pup and return it back to the nest; if the displaced pup was not retrieved within 3 minutes, the pup was returned to the heating pad and the trial was scored as a failure. If the pup was successfully retrieved, the time to retrieve and bring back the pup to the nest zone was scored. After testing all pups of the littermate, pups were placed back into their home cage with their dam. A second session was then run under cool condition (pup’s zone at 17 °C). After each session, virgin female was placed in the nest zone and trials were run with pups. Independent sessions consisted to perform reversal assays by changing the temperature of the pup’s zone (17 °C then 25 °C) in order to exclude any potentiation effect of the dam to retrieve pups. We used an ultrasonic microphone (Avisoft) to verify that isolated pups vocalized during testing.

### Pharmacology treatment

Intranasal administration (IN) was performed in P2 mice 10 min before USV recording or pup’s retrieval assay. Intranasal administration (IN) was performed in P2 mice 10 min before USV recording or pup’s retrieval assay. We used an insulin syringe and smoothed the tip of the needle to avoid injury. The pup is gently handled between two fingers in supine position with the head slightly backwards. The needle is placed in front of the nostril and 10 µl are slowly distributed in order to allow the pup to inhale the solution. Before running all the experiments, we guaranteed that such injections were done reaching the nasal cavity by injecting ink and slicing tissue. The solutions injected were isotonic saline (10 μl) for the control mice and 2 μg of OT (Phoenix Pharmaceuticals Inc., cat #051–01) or 0.2 µg (Thr^4^,Gly^7^)-Oxytocin(TGOT) (BACHEM, lot #1062174) or 0.25 µg of Vasopressin (Phoenix Pharmaceuticals Inc., cat #065–07) diluted in isotonic saline (10 μl) for the treated mice. The intranasal OT, TGOT and AVP doses tested were selected based on previous behavioral studies in mouse. For OT, we used a single acute administration of 2 µg. This dosing is identical to the ones employed for newborns in our previous published work [31, 32, 34]. Such dosing was also used in prairie vole neonate’s publications [35, 36]. For TGOT and AVP, assuming an average weight of 2 g for a P2 mouse, 0.2 µg of TGOT and 0.25 µg of AVP correspond to 0.10 and 0.12 µg/g, respectively. These dosing are in the same range with that employed in previous published behavioral tests employing intranasal administration in adult mice (e.g., 0.05 µg/g) [37].

CNO (Clozapine-N-oxide; Sigma-Aldrich, St Louis, MO, USA) was dissolved in dimethyl sulfoxide (DMSO; Sigma-Aldrich, St Louis, MO, USA) and diluted with 0.9% isotonic saline to volume, the DMSO concentrations in the final CNO solutions were 0.5%. 1 µg of CNO was administrated by subcutaneous route in a total volume of 10 µl. Administration was performed in P2 mice 2 h before USV recording.

### Triglycerides analyses

BATs from P2 pups were extracted and incubated with 500 µL of Chloroform/Methanol (CHCl_3_:CH3OH, 2:1 _*V/V*_) solution. Organic phase was isolated by centrifugation at 10,000 rpm, washed by 0.2 vol of 0.9% NaCl solution, dried over MgSO_4_ and then concentrated under a nitrogen stream.

19 µL of CH_2_Cl_2_ were added per mg of BAT. For TAG analysis, 2 µL of extracts containing total lipids were separated on TLC (Silica Gel 60, Merck) by using petroleum ether:diethyl ether (90:10, *v*/*v*) as eluent. The TLC plates were sprayed with a solution of 5% phosphomolybdic acid in ethanol followed by heating at 120 °C in an oven for 5–10 min, to visualize the spots.

Each resolved plate was scanned using a Chemidoc^TM^ MP Imaging System (Bio-Rad), and densitometric analyses were performed using the ImageLab^TM^ software version 5.0 (Bio-Rad) to determine relative Triglyceride content per sample.

### 2-photon calcium imaging

GG slices of 400 µm were prepared from fresh tissue. Animals were euthanized by decapitation; the nose was dissected and bathed into a vibratome chamber (Leica) containing oxygenated (95% O_2_ and 5% CO_2_) aCSF. aCSF composition was as follows (in mM): 126 NaCl, 3.5 KCl, 2 CaCl_2_, 1.3 MgCl_2_, 1.2 NaH_2_PO_4_, 25 NaHCO_3_ and 11 glucose, pH 7.4 equilibrated with 95% O_2_ and 5% CO_2_. GG slices were incubated with 10 µM of Fura-2-AM (Life technologies) added with Pluronic acid and dissolved in DMSO, for 45 min at 33 °C in an oxygenated artificial cerebrospinal fluid (aCSF) dark chamber. The recording chamber was first filled with warm (30 °C) aCSF for 1 min, then perfused with cool (15 °C) aCSF for 3 min and then warm with aCSF for 1 min. Images were acquired every 5 s with an Olympus BX61WI microscope equipped with a multibeam multiphoton pulsed laser scanning system (LaVision BioTecs) as previously described [38]. Images were acquired through a CCD camera, which typically resulted in a time resolution of 50–150 ms per frame. Slices were imaged using a 20×, NA 0.95 objective (Olympus). Images were collected by CCD-based imaging system running ImspectorPro software (LaVision Biotec) and analyzed with Fiji software [39].

### Protein extraction and western blotting

Brain and brown adipose tissues were homogenized in RIPA buffer (Thermo Fisher Scientific) with phosphatase and protease inhibitor cocktails (Pierce Protease and Phosphatase Inhibitor Mini Tablets, EDTA-Free) added with 1% Triton (Euromedex, life sciences products) for the brown adipose tissues. Proteins were run on a polyacrylamide gel (Bolt 4–12% Bis Tris plus, Invitrogen by Thermo Fisher Scientific) and transferred to a nitrocellulose membrane (GE Healthcare Life Science). Primary antibodies were incubated overnight at 4 °C and were as follows: UCP1 (1:1000, Cell Signalling technology, #14670); p44/42 MAPK (1:1000, Cell Signalling technology, #9102); phospho-p44/42 MAPK (1:1000, Cell Signalling technology, #9101); GAPDH (1:1000, Invitrogen#PA1987). Signals were detected using Super Signal West Pico (Thermo Fisher Scientific, #34080) and bands were analyzed with ImageJ.

### Reverse transcription and real time quantitative PCR

Wild-type and mutant newborns were sacrificed at P2 (between 2 pm and 4 pm). The hypothalamus, BAT and dorsal root ganglia (DRG) tissues were quickly dissected on ice and rapidly frozen in liquid nitrogen, then stored at −80 °C. Total RNA was isolated using the RNeasy® Mini Kit (Qiagen, cat #74104), according to the manufacturer’s protocol and cDNAs were obtained by reverse transcription using QuantiTect® Reverse Transcription Kit (Qiagen, cat #205311), starting with 600 ng of total RNA.

### Statistical analysis

Statistical analyses were performed using GraphPad Prism (GraphPad Software, Prism 8.0 software, Inc, La Jolla, CA, USA). All the statistical analyses are reported in legends. Values are indicated as follows: (Q2 (Q1, Q3) or mean ± SEM, n, p-value, statistical test) where Q2 is the median, Q1 is the first quartile and Q3 is the third quartile.

## Results

### ASD-related Magel2 mutation leads to neonatal thermosensory behavior alterations and impairment of maternal pup retrieval during the first week of life

**Fig. 1:**
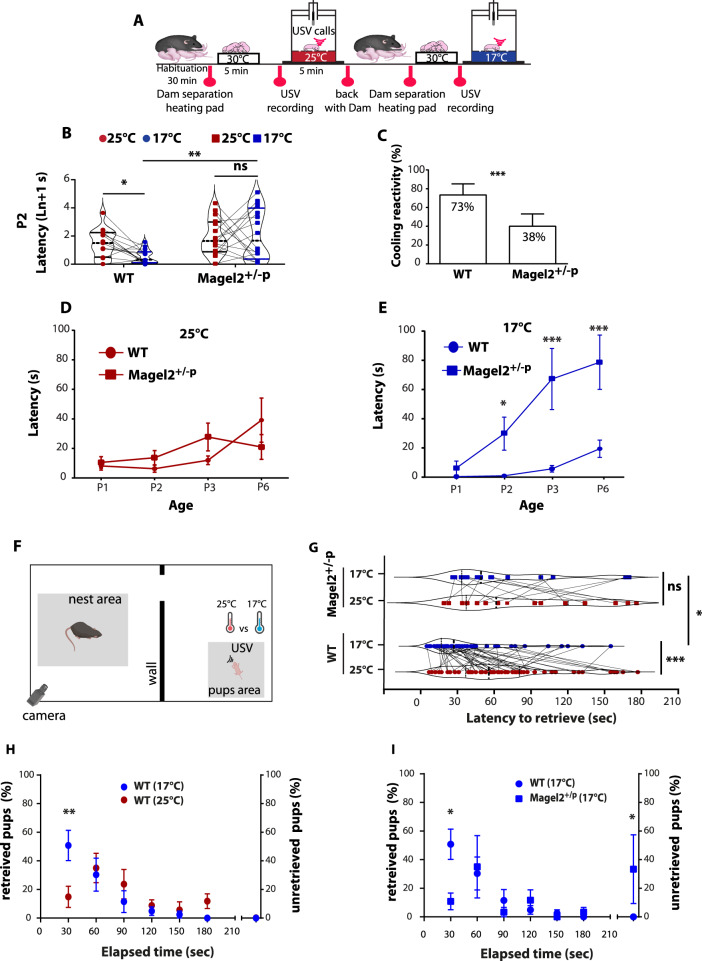
Coolness reactivity failure in *Magel2* deficient neonates. **A** Experimental procedure. After room habituation, neonates are separated from the dam, placed on a heating pad and each neonate is isolated for USVs recording at 25 °C for 5 min. This procedure is repeated a second time at 17 °C exposure, thus values are matched within-subject factor of “temperature”. Such procedure was reconducted from P1 to P6 on WT and Magel2^+/−p^ neonates. **B** Before/after graphs illustrating the latency to the first call measured upon exposure at 25 °C (red dots) followed by 17 °C (blue dots) in WT and in Magel2^+/−p^. WT (25 °C *vs* 17 °C): 1.48 ± 0.26 ln+1 s *vs* 0.55 ± 0.13 ln+1 s, *n* = 15; *p* = 0.03. Magel2^+/−p^ (25 °C *vs* 17 °C): 1.95 ± 0.31 ln+1 s *vs* 2.14 ± 0.45 ln+1 s, *n* = 15; *p* > 0.99. WT *vs* Magel2^+/−p^ (25 °C): 1.48 ± 0.26 ln+1 s, *n* = 15 *vs* 1.95 ± 0.31 ln+1 s, *n* = 15; *p* > 0.99. WT *vs* Magel2^+/−p^ (17 °C): 0.55 ± 0.13 ln+1 s, *n* = 15 *vs* 2.14 ± 0.45 ln+1 s, *n* = 15; p = 0.001. Repeated-measures (temperature) Two-way ANOVA, Bonferroni’s post-test. **C** Bar graphs comparing animals’ responsive rate of coolness-stimulated USV between WT and Magel2^+/−p^ neonates. WT: 73.33 ± 11.82%, *n* = 15 *vs* Magel2^+/−p^: 20 ± 10.69%, *n* = 15, *p* < 0.0001. Fisher’s exact test. **D, E** Comparison of the latencies to the first call over the age between WT and Magel2^+/−p^ neonates at 25 °C (**D**) and 17 °C (**E**). WT *vs* Magel2^+/−p^ (17 °C): P2: WT 0.96 ± 0.28 s, *n* = 15 *vs* Magel2^+/−p^ 29.81 ± 11.32 s, *n* = 15; *p* = 0.03. P3: WT 5.71 ± 2.26 s, *n* = 15 *vs* Magel2^+/−p^ 67.15 ± 20.92 s, *n* = 15, *p* = 0.005. P6: WT 19.47 ± 5.90 s, *n* = 12 *vs* Magel2^+/−p^ 78.69 ± 18.59 s *n* = 12, *p* = 0.007. Repeated-measures (age) Two-way ANOVA, Bonferroni’s post-test. **F** Schematic representation of the Pup Retrieval assay using new cohorts of animals. Values are matched within-subject factor of “temperature”. **G** Latency of WT dams to retrieve their WT or Magel2^+/−p^ pups under room temperature (25 °C) and Cool environment (17 °C). WT 25 °C: 69.23 ± 5.94 s *vs* WT 17 °C: 41.02 ± 4.71 s, *n* = 53; *p* = 0.0009. Magel2^+/−p^ 25 °C: 83.68 ± 13.27, *n* = 16 *vs* Magel2^+/−p^ 17 °C: 70.56 ± 11.69, *n* = 16; *p* = 0.5754. WT 25 °C: 69.23 ± 5.99 s, *n* = 53 *vs* Magel2^+/−p^ 25 °C: 83.68 ± 13.27 s, *n* = 16; *p* = 0.45. WT 17 °C: 41.02 ± 4.71 s, *n* = 53 *vs* Magel2^+/−p^ 17 °C: 70.56 ± 11.69 s, *n* = 16; *p* = 0.0287. Repeated-measures (temperature) Two-way ANOVA, Bonferroni’s post-test. **H, I** Percentage of WT and Magel2^+/−p^ pups retrieved by their respective WT dam over the time course of the Pup Retrieval assay. On the right side of each graph the proportion of non-retrieve pups is indicated. WT pups retrieval between 25 °C and 17 °C environmental conditions: WT 25 °C: 14.86 ± 7.41; *n* = 39 *vs* WT 17 °C: 50.79 ± 10.58; *n* = 39; *p* = 0,0066; Repeated-measures (temperature) Two-way ANOVA, Bonferroni’s post-test (**H**). Pups retrieval between WT and Magel2^+/−p^ pups under 17 °C: WT: 50.79 ± 10.58 *vs* Magel2^+/−p^:^:^ 10.83 ± 5.83, *n* = 26; *p* = 0,0464. WT unretrieved: 0.2 ± 0.2; *n* = 39 *vs* Magel2^+/−p^ unretrieved: 33.33 ± 24.04; *n* = 26; *p* = 0,0338; Two-way ANOVA, Bonferroni’s post-test (**I**). Among the proportion of unretrieved Magel2^+/−p^, no significant difference was observed between male and female. Magel2^+/−p^ male: 29%; *n* = 17; Magel2^+/−p^ female: 38%; *n* = 13; *p* = 0.70; Fisher’s exact test. Data are presented as mean ± SEM. *p < 0.05; **p < 0.01; ***p < 0.001; ns Non-significant.

To assess thermosensitivity in neonates, we developed an experimental procedure based on coolness-induced USV [17] (Fig. 1A). Wild-type (WT) and *Magel2*^*+/−p*^ *(*i.e., *Magel2 KO)* neonates aged from 0 to 6 days old (P0 to P6) were taken from their nests, isolated from the dam, and exposed separately and successively to two different temperatures (ambient: 25 °C and cool: 17 °C). Analyzing the latency in emitting the first call, which reflects the reactivity of the animals to sense cold, we found that WT neonates presented a lower latency at cool temperatures than under ambient exposure, while *Magel2*^*+/−p*^ did not (Fig. 1B and Supplemental Fig. 1A, C, E).

Furthermore, the proportion of neonates responsive to cooling was markedly decreased in *Magel2*^*+/−p*^ from P1 to P6 compared to the WT (Fig. 1C and Supplemental Fig. 1B, D, F). Comparison of the latencies (Fig. 1D, E) between WT and *Magel2*^*+/−p*^ revealed a significant age-dependent difference under cool (Fig. 1E) but not ambient exposure (Fig. 1D). We found that this atypical sensory reactivity was not the result of a motor/vocalization deficit since the number of USV and latency of the first calls were not affected under ambient temperature condition (Fig. 1D and Supplemental Fig. 1G). However, the number of calls were significantly reduced under cool exposure (Supplemental figure H). This deficit was independent of the sex (Supplemental Fig. 1G, H). We also found that dam separation and handling did not affect corticosterone levels of *Magel2*^*+/−p*^ and WT in both male and female and cannot be linked to this atypical thermosensory reactivity (Supplemental Fig. 2A, B).

To exclude any potentiation of dam separation, we ran assays in which USV box temperature was kept at 25 °C during the two periods of USV recording (Supplemental Fig. 3A). Using new cohorts of animals, we found that WT performed comparably upon repetitive ambient temperature exposures (Supplemental Fig. 3B, C, G, H). Moreover, we ran experimental paradigm inversion in which another cohort of animals was first exposed to 17 °C and then to 25 °C (Supplemental Fig. 3D). WT neonates still presented a low-latency response when exposed first to cool *versus* ambient temperatures (Supplemental Fig. 3D, E, F, G, H).

Thus, the latency of the first call of isolated neonates was dependent on external temperature. As previously observed, we confirmed that USVs responses to a cool challenge decreased as the pups matured [40]. At P6, only half of the WT animals were reactive upon cool exposure (Supplemental Fig. 1E, F). This responsiveness to cool stimulus declines towards development, which correlates with fur growth, opening of ear canals and the increasing capability of rodents to develop other thermoregulatory capabilities such as seeking comfortable temperature [11–13, 41].

Cool stimulus is a factor that elicits pups USVs allowing mothers to retrieve them [11–13]. We thus assessed the consequences of cool sensitivity alteration on mother-infant interaction. We conducted a behavioral test of pup retrieval under same temperature conditions and by separating the nest from the isolated pup by a wall in a way that the dam can only ear and probably smell the pups (Fig. 1F). It is important to note that both WT and *Magel2*^*+/−p*^ pups (P2) were born and raised by a WT dam. On analyzing the latency of dams to retrieve pups, we found that dams raising WT pups were significantly faster at cool than under ambient temperature to retrieve them, while dams raising *Magel2*^*+/−p*^ pups did not (Fig. 1G). Moreover, under cool condition dams raising WT pups exhibited shorter latency to retrieve their WT pups compared to those raising *Magel2*^*+/−p*^ pups (Fig. 1G). To analyze more finely the reactivity of the dams to retrieve their pups, we measured every 30 s throughout the test the proportion of retrieved pups. We observed that less than 15% of WT pups were retrieved during the first 30 seconds of the test in ambient condition while under cool condition this proportion was significantly increased to 50% (Fig. 1H). In contrast, at the same time, the proportion of retrieved *Magel2*^*+/−p*^ pups under cool temperature was only 10% (Fig. 1I). Furthermore, more than a third of *Magel2*^*+/−p*^ pups were not retrieved which was not observed for WT pups in cool condition (Fig. 1I). We found no difference between male and female of those unretrieved *Magel2*^*+/−p*^ (Fig. 1I).

### Cool thermo-sensory behavior impairment in Magel2^+/−p^ neonates is not linked to a deficiency in non-shivering thermogenesis

**Fig. 2:**
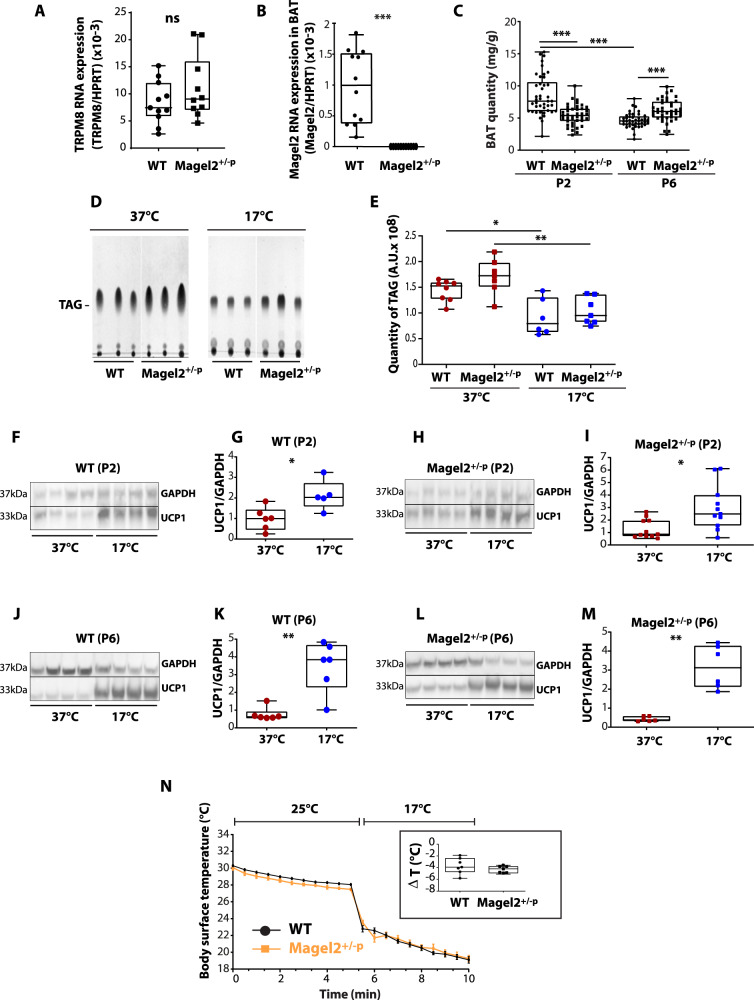
TRPM8 and brown adipose tissue investigations after cool exposure in WT and *Magel2*^*+/−p*^. **A** mRNA expression of TRPM8 in DRG. Quantification of TRPM8 RNA transcripts in DRG of WT and Magel2^+/−p^ at P2. WT: 0.007 (0.005; 0.012); *n* = 11 *vs* Magel2^+/−p^: 0.009 (0.007, 0.016); *n* = 10; *p* = 0.34; Mann Whitney test. Data are presented as median (with interquartile range). **B** Quantification of Magel2 RNA transcripts in WT and Magel2^+/−p^ in BAT at P2. WT: 1.03 × 10^−^^3^(4.20 × 10^−4^; 1.53 × 10^−3^); *n* = 12 *vs* Magel2^+/−p^: 0 (0); *n* = 11; *p* < 0.0001. Mann Whitney test. **C** Brown adipose tissue (BAT) weight normalized to the body weight of WT and Magel2^+/−p^ at P2 and P6. P2: WT 8.52 ± 0.47 mg/g; *n* = 42 *vs* Magel2^+/−p^: 5.25 ± 0.26 mg/g; *n* = 40; *p* < 0.0001; P6: WT: 4.66 ± 0.17 mg/g, *n* = 42 *vs* Magel2^+/−p^: 6.18 ± 0.27 mg/g, *n* = 40, *p* = 0.0045. Two-way ANOVA, Bonferroni’s post-test. **D, E** Total lipid extraction of WT and Magel2^+/−p^ BAT and thin layer chromatography analysis of TAG (**D**). Quantifications of TAG (**E**): WT 37 °C: 1.44.10^8^ ± 0.07.10^8^, *n* = 8 *vs* WT 17 °C: 0.91.10^8^ ± 0.14.10^8^ *n* = 6; *p* = 0.04. Magel2^+/−p^ 37 °C: 1.70.10^8^ ± 0.13.10^8^, *n* = 7 *vs* Magel2^+/−p^ 17 °C: 1.03.10^8^ ± 0.10.10^8^ *n* = 7; *p* = 0.005. Two-way ANOVA, Bonferroni’s post-test. **F**–**M** Immunoblot analyses and quantifications of UCP1 expression after cool exposure at P2 (**F**–**I**) in WT and Magel2^+/−p^ (**F**, **G** and **H**, **I** respectively) and at P6 (**J**–**M**) in WT and Magel2^+/−p^ (**J**, **K** and **L**, **M** respectively). **G** WT P2 :0.99 (0.48, 1.41), *n* = 6, *vs* 2.03 (1.61, 3.25), *n* = 5, *p* = 0.0173; **I** Magel2^+/−p^ P2: 0.86 (0.71, 1.95), *n* = 11, *vs* 2.49 (1.56, 4), *n* = 11, *p* = 0.0104. K: WT P6: 0.62 (0.57; 0.89), *n* = 6, *vs* 3.84 (2.31; 4.64), *n* = 6, *p* = 0.0043; **M** Magel2^+/−p^ P6: 0.38 (0.32; 0.58), *n* = 6, *vs* 3.12 (2.11; 4.29), *n* = 6, *p* = 0.0022. Mann Whitney test. **N** Time course of loss of surface body skin temperature in WT (black line) and Magel2^+/−p^ (orange line) of P2 neonates during a temperature challenge (5 min at 25 °C then 5 min at 17 °C). Data are presented as mean ± SEM. Insert represents the delta loss of surface body temperature before (at 5 min) and after cool exposure (at 10 min) in WT and Magel2^+/−p^ P2 neonates. WT: −3.9 (−4.7; −2.4), *n* = 7 *vs* Magel2^+/−p^: −4.2 (−4.9; −3.8), *n* = 7, *p* = 0.3648. Mann Whitney test. Data are presented as median (with interquartile range).

We first supposed that this neonatal atypical thermo-sensory response could be linked to non-shivering thermogenesis dysregulation involving peripheral thermoreceptors, namely TRPM8, expressed in sensory neurons of the DRG [42]. *Magel2* being expressed in the DRG [43, 44], we first analyzed whether peripheral expression of TRPM8 could be affected in *Magel2*^*+/−p*^ compared to WT neonates. We found no significant difference of the quantity of TRPM8 transcripts in DRG neurons (Fig. 2A).

We found that *Magel2* is expressed in BAT (Fig. 2B) and that P2 *Magel2*^*+/−p*^ neonates had significant decreased interscapular BAT mass compared to age-matched WT (Fig. 2C). WT BAT mass was developmentally reduced from P2 to P6. Such decline was not observed in mutants. Thus, at P6, *Magel2*^*+/−p*^ neonates had significant increased interscapular BAT mass compared to age-matched WT (Fig. 2C). The difference in the amount of BAT could suggest thermogenesis dysregulation, but previous study has demonstrated that acute non-shivering thermogenesis is independent of BAT mass [45]. Thus, in order to measure non-shivering thermogenesis activity, we extracted P2 interscapular BAT tissues after 1 h exposure to cool temperature (17 °C) and analyzed BAT lipolysis and expression of the uncoupling protein 1 (UCP1), a mitochondrial protein activity marker from BAT responsible for non-shivering thermogenesis [46]. Quantitative analyses of BAT lipids, separated by thin layer chromatography (Fig. 2D), show that cold exposure induced a similar significant consumption of triglycerides (TAG) in both WT and *Magel2*^*+/−p*^ pups (Fig. 2E). Furthermore, we found that upon acute cool exposure (17 °C, 1 h), UCP1 protein expression significantly increases in P2 WT (Fig. 2F, G) as well as *Magel2*^*+/−p*^ (Fig. 2H, I). Similar results were observed at P6 (Fig. 2J–M).

Thus, these results demonstrate that UCP1-mediated non-shivering thermogenesis in BAT is fully active in *Magel2*^*+/−p*^. They are also consistent with recent findings showing that UCP1 activation is independent of BAT mass [45]. We finally followed skin body temperatures upon cool temperature challenge and found that temperature of *Magel2* deficient neonates drop similarly as WT (Fig. 2N).

Altogether, our results demonstrate that lack of pup calls reactivity to cool sensory stimuli found in *Magel2* deficient neonates is related to their capacity of regulating temperature.

### Cool thermo-sensory behavior impairment in Magel2^+/−p^ neonates is not linked to dysfunction of the peripheral thermosensitive neurons from the Grueneberg ganglion

**Fig. 3:**
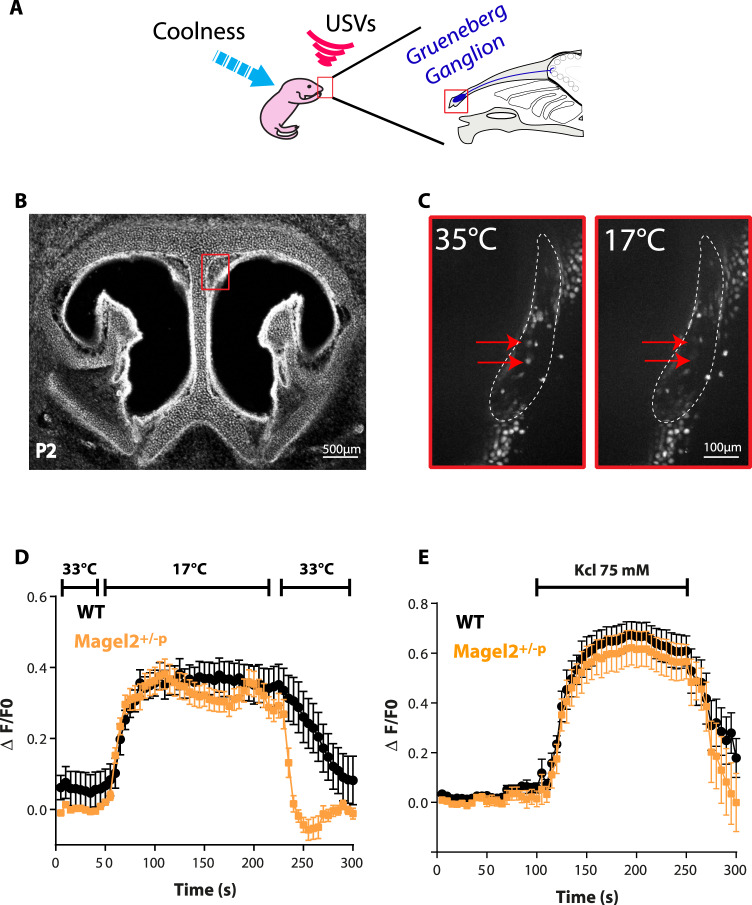
Coolness-induced response in the Grueneberg Ganglion of *Magel2*^*+/−p*^. **A** Schematic representation of the role and localization of the GG. **B** Coronal sections of the nasal cavity of a P2 neonate with localization of the GG (red box). **C** Inserts represent calcium imaging responses after cool exposure. **D** Ca^2+^ signals induced by cooling from 35 °C to 17 °C in WT (*n* = 8) and Magel2^+/−p^ (*n* = 9) GG neurons (respectively black and orange). **E** Ca^2+^ signals induced in GG neurons by perfusion with KCl (75 mM) were used as a control for viability and responsiveness of tissues slices in WT (*n* = 10) and Magel2^+/−p^ (*n* = 11) GG neurons. ΔF represents change in the ratio of the fluorescence intensity measured in cells; Data are represented as mean ± SEM.

Peripheral perception to cool temperature is also conducted by the Grueneberg Ganglion (GG), a sensory organ located at the tip of the nose (Fig. 3A). This ganglion contains sensitive neurons responding to cool temperatures [18] and it has been proposed to influence USV [20] generated by rodent neonates to elicit maternal care under cool temperature exposure [11, 13, 21]. Interestingly, neonate mice deleted for the thermoreceptor expressed in these sensory neurons present USV calls impairment after cool exposure; a phenotype very similar to what we observe here in *Magel2*^*+/−p*^ [17]. We thus ask whether dysfunction of these peripheral thermo-sensory neurons might be affected in *Magel2*^+/−p^. We conducted 2-photon calcium imaging and single cell analyses on tissue slices through the GG of P2 neonates (Fig. 3B, C). Thermo-evoked neuronal activities (obtained by decreasing the temperature of the perfusion solution from 35 °C to 17 °C) elicited a substantial increase in intracellular Ca^2+^ in both all WT and *Magel2*^*+/−p*^ animals tested (Fig. 3D, E).

Thus, the thermosensory neurons of the GG are functional in the *Magel2*^*+/−p*^ neonates.

### Neonatal inactivation of oxytocinergic neurons in WT mimics cool thermo-sensory behavior impairment found in Magel2^+/−p^

**Fig. 4:**
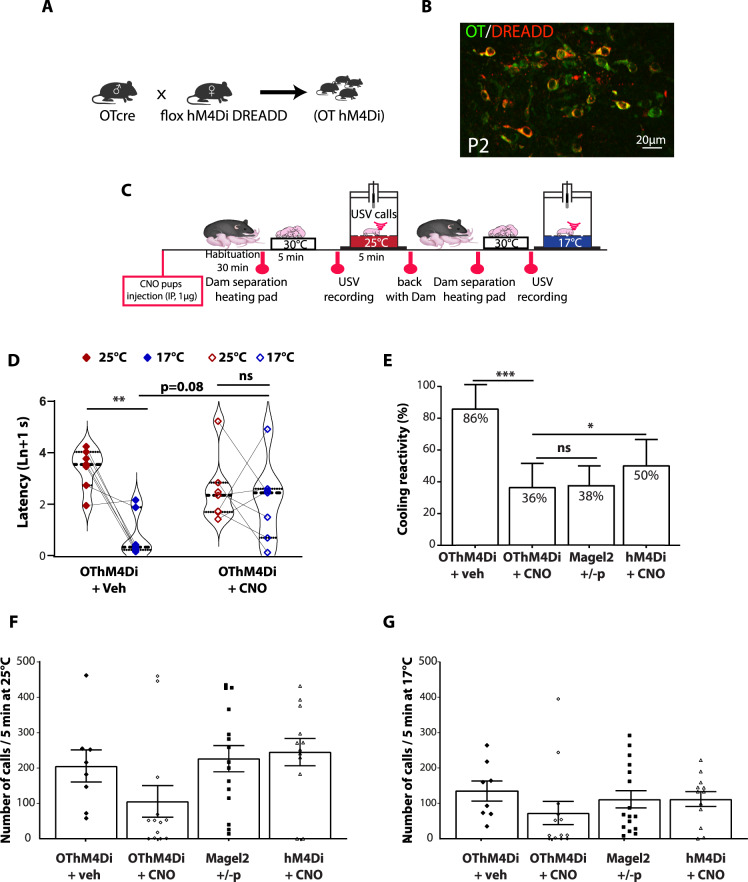
Coolness reactivity failure in WT after oxytocinergic neurons inactivation. **A** Generation of the hM4Di DREADD OTCre mice (OThM4Di). **B** Immunohistochemistry illustrating the expression of hM3Di (red) in OT neurons (green). **C** Experimental procedure: IP injection of CNO (1 µg) or vehicle was performed in two independant groups of P2 OThM4Di neonates 2 h before starting experiment. After room habituation, neonates are separated from the dam, placed on a heating pad and each neonate is isolated for USVs recording at 25 °C for 5 min. This procedure is repeated a second time at 17 °C exposure, thus values are matched within-subject factor of “temperature”. **D** Before/after graphs illustrating the latency to the first call measured upon exposure at 25 °C (red dots) followed by 17 °C (blue diamonds) in neonates expressing the hM4Di receptor (OThM4Di) treated with vehicle (Veh) or with CNO. Veh 25 °C: 3.39 ± 0.3; *n* = 7 *vs* Veh 17 °C: 0.78 ± 0.32; *n* = 7; *p* = 0.001. CNO 25 °C: 2.53 ± 0.48; *n* = 7 *vs* CNO 17 °C: 2.11 ± 0.60; *n* = 7; *p* = 0.84. Veh 25 °C: 3.39 ± 0.3; *n* = 7 *vs* CNO 25 °C: 2.53 ± 0.48; *n* = 7; *p* = 0.36. Veh 17 °C: 3.39 ± 0.3; *n* = 7 *vs* CNO 17 °C: 2.53 ± 0.48; *n* = 7; *p* = 0.08. Repeated-measures (temperature) Two-way ANOVA, Bonferroni’s post-test. **E** Responsive rate of coolness-induced USV in OThM4Di neonates treated with vehicle or CNO (85.71 ± 14.29%, *n* = 7 *vs* 36.36 ± 15.21%, *n* = 11; *p* < 0.0001). Cooling reactivity of OThM4Di neonates treated with CNO was also compared with either Magel2^+/−p^ (37.5 ± 12.5%, *n* = 16; *p* = 0.1169) or neonates non-expressing the hM4Di receptor (hM4Di) (50 ± 16.67 ln+1 s, *n* = 10, *p* = 0.0315). Fischer’s exact test. **F, G** Total number of calls at 25 °C (**F**) and 17 °C (**G**) in OThM4Di neonates treated or not with CNO and compared with either Magel2^+/−p^ or neonates non-expressing the hM4Di receptor (hM4Di). **F** OThM4Di (veh *vs* CNO): 205 ± 45, *n* = 8 *vs* 105 ± 44, *n* = 13; *p* = 0.5. OThM4Di+CNO: 105 ± 44, *n* = 13 *vs* Magel2^+/−p^: 227 ± 37, *n* = 15; *p* = 0.15. OThM4Di+CNO: 105 ± 44, *n* = 13 *vs* hM4Di+CNO: 245 ± 39, *n* = 12; *p* = 0.07. **G** OThM4Di (veh *vs* CNO): 134 ± 28, *n* = 8 *vs* 72 ± 32, *n* = 13; *p* = 0.18. OThM4Di+CNO: 72 ± 32, *n* = 13 *vs* Magel2^+/−p^: 111 ± 24, *n* = 16; *p* = 0.53. OThM4Di+CNO: 72 ± 32, *n* = 13 *vs* hM4Di+CNO: 111 ± 20, *n* = 12; *p* = 0.52. Kruskal-Wallis test, Dunn’s post-test. Data are presented as mean ± SEM.

OT is known to regulate an individual’s adaptation to the environment [47], largely by modulating sensory systems [29]. To directly address the involvement of OT neurons in neonatal thermosensory reactivity, we assessed whether inactivation of OT neurons of WT neonates can mimic thermosensory impairment observed in *Magel2*^*+/−p*^ using DREADD (Designer Receptors Exclusively Activated by Designer Drugs) technology [48]. These DREADD can be activated by the ligand clozapine N-oxide (CNO) and its metabolite, clozapine; both drugs crossing the BBB [49]. We restricted hM4Di-mCherry expression to OT neurons by crossing hM4Di-mCherry mice (named here hM4Di) with OT Cre (Fig. 4A, B). These mice were called here OThM4Di.

Vehicle or CNO (1 µg) was injected into P2 neonates by intraperitoneal (IP) administration 2 h before starting thermo-sensory behaviors (Fig. 4C). We found that vehicle-treated OThM4Di animals, presented a significant faster reaction in emitting their first call when exposed to cool *versus* ambient temperatures (Fig. 4D); while CNO-treated OThM4Di did not (Fig. 4E). Furthermore, the animal responsive rate to cool temperature was markedly decreased in CNO-treated OThM4Di with percentages reaching similar values than *Magel2*^*+/−p*^ neonates (Fig. 4E). CNO treatment did not affect the numbers of USV calls of OThM4Di neonates either at ambient (25 °C) or cool temperature (17 °C) (Fig. 4F, G).

Thus, our results reveal that in vivo inactivation of OT neurons prevents neonates to respond to cool temperatures and suggest that the OT system can regulate call reactivity behavior of neonates being exposed to cool temperature.

### Intranasal injection of oxytocin and oxytocin receptor agonists rescue cool sensitivity call behavior in Magel2^+/−p^ neonates and potentiates maternal pup retrieval

**Fig. 5:**
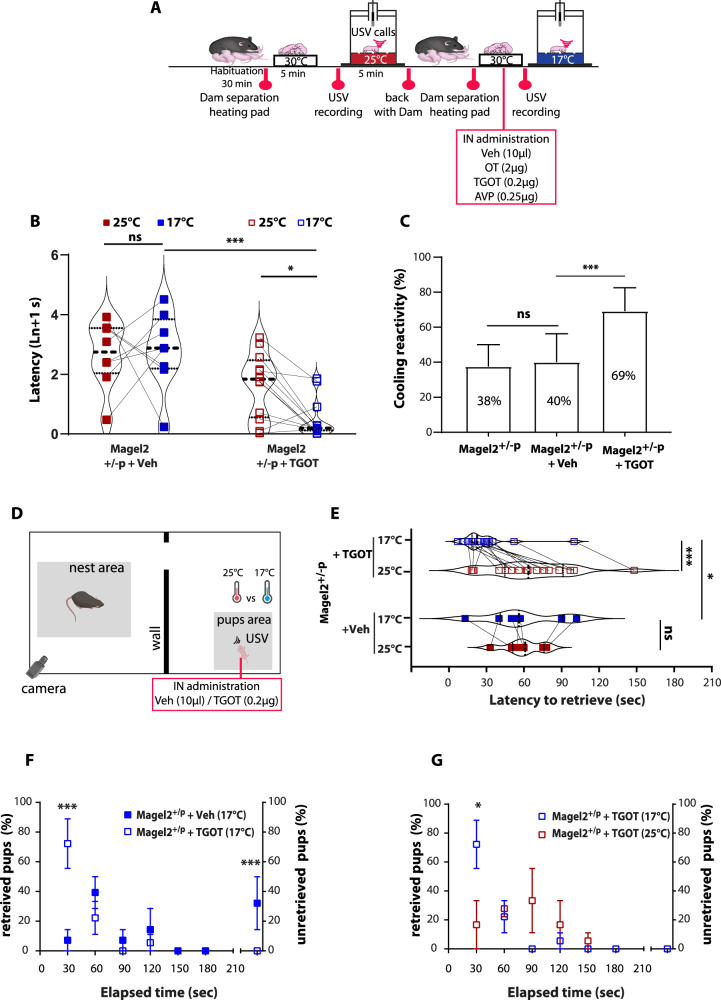
Intranasal oxytocin and oxytocin receptor agonists rescue coolness reactivity in *Magel2*^*+/−p*^. **A** Experimental procedure. After room habituation, 4 independent groups (treatments) of neonates are separated from the dam, placed on a heating pad and each neonate is isolated for USVs recording at 25 °C for 5 minutes. 10 minutes before recording the USVs at 17 °C, neonates received an intranasal injection (IN) of Vehicle (Veh, 10 µl NaCl), or Oxytocin (OT, 2 µg) or (Thr4,Gly7)-Oxytocin (TGOT, 0.2 µg) or Vasopressin (AVP, 0.25 µg). Values are matched within-subject factor of “temperature”. **B** Before/after graphs represent the latency to the first call measured at 25 °C (red squares) and 17 °C (blue squares) in Magel2^+/−p^ treated with vehicle (Veh) or TGOT. TGOT 25 °C: 1.65 ± 0.31 ln+1 s vs TGOT 17 °C*:* 0.5 ± 0.19 ln+1 s, *n* = 12; *p* = 0.02. Veh 25 °C: 2.66 ± 0.39; *n* = 8 *vs* TGOT 25 °C: 1.65 ± 0.31 ln+1 s; *n* = 12; *p* = 0.08. Veh 17 °C: 2.79 ± 0.46 ln+1 s, *n* = 8 vs TGOT 17 °C: 0.5 ± 0.19 ln+1 s, *n* = 12; *p* = 0.0005. Repeated-measures (temperature) Two-way ANOVA, Bonferroni’s post-test. **C** Bar graphs showing animals responsive rate of coolness-stimulated USV in Magel2^+/−p^ untreated, vehicle (Veh) or TGOT treated conditions. Magel2^+/−p^ untreated: 38 ± 12% *n* = 16 *vs* Magel2^+/−p^ + Veh: 40 ± 16%, *n* = 9; *p* = 0.77. Magel2^+/−p^ + Veh: 40 ± 16% *n* = 9 *vs* Magel2^+/−p^ + TGOT: 69 ± 13%, *n* = 13; *p* < 0.0001. Fisher’s exact test. **D** Schematic representation of the Pup Retrieval assay using new cohorts of animals.Two independant groups (Vehicle or TGOT) of Magel2^+/−p^ are used and values are matched within-subject factor of “temperature”. **E** Latency of WT dams to retrieve their Magel2^+/−p^ pups treated with TGOT or with a vehicle solution (Veh) under room temperature and Cool environment. Veh (25 °C): 58.71 ± 5.75 *vs* Veh (17 °C): 58.14 ± 11.32; *n* = 7; *p* > 0.99. TGOT (25 °C): 65.66 ± 7.91 *vs* TGOT (17 °C): 27.72 ± 4.89; *n* = 18; *p* < 0.001. Veh (25 °C): 58.71 ± 5.75; *n* = 7 *vs* TGOT (25 °C): 65.66 ± 7.91; *n* = 18; *p* > 0.99. Veh (17 °C): 58.14 ± 11.32; *n* = 7 *vs* TGOT (17 °C): 27.72 ± 4.89; *n* = 18; *p* = 0.029. Repeated-measures (temperature) Two-way ANOVA, Bonferroni’s post-test. **F, G** Percentage of Magel2^+/−p^ pups treated with intranasal TGOT injection (0.2 µg) or with a vehicle solution (Veh) and retrieved by their WT dam over the time course of the Pup Retrieval assay. On the right side of each graph the proportion of non-retrieve pups is represented. Pups retrieval between Magel2^+/−p^ + Veh and Magel2^+/−p^ + TGOT pups under 17 °C: Magel2^+/−p^ + Veh: 7.14 ± 7.14; *n* = 9 *vs* Magel2^+/−p^ + TGOT^:^ 72.23 ± 16.67, *n* = 18; *p* < 0.001. Magel2^+/−p^ + Veh unretrieved: 32.14 ± 17.86; *n* = 9 *vs* Magel2^+/−p^ + TGOT unretrieved: 0 ± 0; *n* = 18; *p* = 0,0153; Two-way ANOVA, Bonferroni’s post-test. **F** Comparison of Magel2^+/−p^ + TGOT pups retrieval between 25 °C and 17 °C. Magel2^+/−p^ + TGOT (25 °C): 16.67 ± 16.67 *vs* Magel2^+/−p^ + TGOT (17 °C): 72.23 ± 16.67; *n* = 18; *p* < 0.001; Repeated-measures (temperature) Two-way ANOVA, Bonferroni’s post-test (**G**). Data are presented as mean ± SEM.

We ask whether pharmacological OT treatment could improve thermosensory call behavior in *Magel2*^*+/−p*^ during the neonatal period (P2). Of the two preferred routes to reach the cerebrospinal fluid, and considering the small size of neonate mice, we found more appropriate to administrate OT by intranasal (IN) rather than intravenous route [50, 51]. New cohorts of neonatal mice were tested for cool thermosensory call behavior with a similar procedure except that those neonates received the treatment between ambient and cool exposures. This procedure allows us to analyze the effect of an acute OT treatment by performing paired comparison between ambient *versus* cool exposure responses within a same animal (Fig. 5A).

We first verified that handling and IN administration procedures did not affect cool-induced call behavior of *Magel2*^*+/−p*^ neonates. After vehicle treatment (saline solution), *Magel2*^*+/−p*^ were still unable to react to cool exposure since the latency to the first call under cool exposure was similar to ambient exposure (Fig. 5B). Furthermore, comparison of the responsive rate to cool temperature (i.e. the proportion of neonates responsive to cooling) under cool exposure revealed an insignificant change between Magel2^+/−p^ untreated and vehicle treated groups (Fig. 5C).

We found that IN administration of OT (2 µg) significantly decreased the latency of the first call of *Magel2*^*+/−p*^ neonates under cool exposure (Supplemental Fig. 4A) and markedly increased the responsive rate of *Magel2*^*+/−p*^ (Supplemental Fig. 4B). Indeed, after OT injection, 77% of *Magel2*^*+/−p*^ neonates reacted to cool stimuli ((Supplemental Fig. 4B), a percentage similar to the P2 WT (Fig. 1C). Thus, an OT pharmacological treatment can rescue the cool thermosensory call behavior deficit of the *Magel2*^*+/−p*^ neonates.

To better characterize the pathway implicated in the rescue of the cool-induced call behavior, we tested two OT receptor agonists: vasopressin (AVP) which activate the OT receptor with the same affinity as OT and [Thr^4^,Gly^7^]OT also referred to as TGOT, a selective OT receptor agonist [52, 53]. We thus treated *Magel2*^*+/−p*^ neonates (P2) with either TGOT or AVP and performed USV call recording 10 min after drug administration. By analyzing the reactivity of the animals to sense cool temperature, we found that *Magel2*^*+/−p*^ neonates (P2) presented a significant faster reaction in emitting their first call when exposed to cool *versus* ambient temperature after either TGOT (Fig. 5B); the difference between treatment (+Veh versus +TGOT) under cool exposure being also significant (Fig. 1B). Similar results were obtained with AVP treatment (Supplemental Fig. 4C). Furthermore, the responsive rate of *Magel2*^*+/−p*^ neonates to cool temperature was markedly increased in both TGOT (Fig. 5C) and AVP conditions (Supplemental Fig. 4D); reaching similar values as the P2 WT (Fig. 1C). We also addressed the action of TGOT in WT neonates and found that treatment preserved both the response and the number of USV upon cool temperature exposure (Supplemental Fig. 4F, H).

Finally, *Magel2*^*+/−p*^ pups treated either with AVP or TGOT evoked substantial USV call number upon cool exposure reaching values similar to P2 WT (Supplemental Fig. 4G). Although we cannot completely exclude a minor contribution of the AVP receptors, these data suggest that the improvement of cool thermosensory call behavior is mainly due to activation of the OT receptors.

Since cool exposure or stress alters the Erk pathways in the brain by reducing Erk activation [54, 55] and OT has been shown to block this alteration [54], we examined whether these signaling pathways might be altered in *Magel2*^*+/−p*^ brain neonates. Erk/P-Erk levels were measured from P2 whole brains of WT and *Magel2*^*+/−p*^ immediately after ambient or cool exposure. Cytoplasmic levels of P-Erk revealed that brain of WT neonates had a significant cool-induced reduction of P-Erk (Supplemental Fig. 5A, C); while *Magel2*^*+/−p*^ did not (Supplemental Fig. 5B, D). More importantly, IN administration of OT allowed a cool-induced reduction of P-Erk in the *Magel2*^*+/-p*^ neonates (Supplemental Fig. 5F, H) without affecting the reduction of P-Erk in the brain of WT (Supplemental Fig. 5E, G).

Thus, these results highlight a deficit in *Magel2*^*+/−p*^ brain development and reveal that OT’s ability to reverse cool thermosensory call behavior may act, at least partly, through Erk pathways.

Considering these results, the fact that OT of mother’s brain is an important modulator of the mother-infant bound [33, 56] and that *Magel2*^*+/−p*^ pups present a dysregulation of the oxytocinergic system [31, 32], we hypothesized that OT of pup’s brain could be also implicated in this bound. By treating *Magel2*^*+/−p*^ pups with TGOT (0.2 µg, *IN*), we found an improvement of dam’s performance to retrieve *Magel2*^*+/−p*^ pups under cool exposure compared to ambient condition (Fig. 5E); as observed in WT pups (Fig. 1G). This result was not observed in vehicle-treated *Magel2*^*+/−p*^ pups (Fig. 5E). By measuring every 30 s throughout the test the proportion of retrieved pups, we found that while only 7% of vehicle-treated *Magel2*^*+/−p*^ pups were retrieved, more than 70% of them were retrieved after TGOT treatment (Fig. 5F). At the end of the test, the proportion of unretrieved *Magel2*^*+/−p*^ pups was also significantly reduced by TGOT treatment since all pups were retrieved under cool condition (Fig. 5F). Finally, on analyzing the effect of TGOT treatment under ambient condition, we observed that during the first 30 s the proportion of retrieved pups was around 15%, while at the end of the test all TGOT-treated *Magel2*^*+/−p*^ were retrieved (Fig. 5G).

Thus, these results demonstrate that maternal pup retrieval is enhanced after administration of the OT receptor agonist in *Magel2*^*+/−p*^.

## Discussion

ASD research has mainly focused on ASD-related genes and their impact on social and cognitive behavior in adult. However, atypical sensory reactivity that represents early markers of autism and are predictive of social-communication deficits and repetitive behaviors in childhood has been largely overlooked. Although recent findings performed in mouse ASD genetic models report sensory deficits [2–8], they were explored during juvenile or adult period. Whether sensory dysfunctions might be present at the early life stage is still unknown.

Here we provide the first experimental evidence that newborn harboring deletion in *Magel2*, an autism associated*-*gene implicated in Prader-Willi and Schaaf-Yang syndromes, exhibit atypical thermosensory behavior during the first postnatal week.

With the aim to investigate any impairment in physiological thermogenesis, we found BAT activation upon cool challenge, suggesting that the autonomic neural circuit controlling non-shivering thermogenesis is not affected and cannot be incriminated for this atypical thermosensory behavior.

Furthermore, functional investigation of the GG revealed that this peripheral cool sensor is still active in *Magel2*^*+/−p*^ neonates.

*Magel2*^*+/−p*^ neonates might encounter difficulties in integrating thermo-sensory stimuli and we provided some evidence for such deficits. First, we can rule out a motor/vocalization deficit since USVs calls were unaffected under ambient temperature at this early developmental stage. We found a lack of cool-induced alteration of brain P-Erk signaling. Second, we showed that brain inactivation of OT neurons in WT reproduces atypical thermo-sensory reactivity. Third, intranasal injection of OT or its agonists can improve thermo-sensory reactivity through activation of the OT receptor. Whilst it is well known that OT plays a major role in modulating sensory systems [29], this modulation could be also indirect through an arousal effect of OT.

Prader-Willi and Schaaf-Yang babies present sensory disorders characterized by temperature instabilities manifested with episodes of hyper or hypothermia without infectious causes [26, 27]. Moreover, adolescent with ASD present loss of sensory function of thermal perception [28]. Here we demonstrate a new pivotal role of the oxytocinergic system in modulating early life thermosensory function that could be involved in these symptoms.

This hypo-thermosensory reactivity of the *Magel2*^*+/−p*^ neonates to cool environment is repercussed on the reactivity of the mother to retrieve their pups. We have demonstrated the existence of a substantial proportion of unretrieved Magel2^+/−p^ pups by their WT dams as well as a delayed latency to retrieve the rest of them under cool exposure. Deficit in maternal care of Magel2^+/−p^ has been reported recently to occur specifically at P8 but not P6 [57]. Whilst they used a different Magel2 mutant mouse model (Magel2^tm1Stw^) and a distinct experimental approach, the difference in age is also an important factor. Therefore, it is possible that maternal care evolves during age pup being more an indicator of danger (here temperature drop) during the first week of life and after an indicator of an affective state.

OT agonist treatment of Magel2^+/−p^ neonates improved both the proportion and the latency to retrieve. This suggests that the oxytocinergic system at P2 modulates the reactivity of newborns to rapidly generate USVs which has a direct effect on mother’s behavior. OT and USVs calls play an important role in mother-infant recognition [29, 58] and they are both dysregulated in this mouse autistic model. Our present results highlight that not only OT from the dam, but also OT from the pups is important in this mother-infant recognition.

Although cool-induced cry has been also observed in newborn infants more than 20 years ago [59], it is rarely observed nowadays because maintaining the body temperature of the neonates has been emphasized. Atypical early vocalization has been detected in 6-month-old infants at risk for autism [14] and may represent an early biomarker for ASD [15]. Measures of early life sensory behavior such as cool-thermosensory call behavior might represent promising avenues for early diagnostic and OT treatment could be considered for therapeutic interventions of this atypical sensory reactivity.

## Supplementary information

Supplemental methods and figures
