# Supplementary material for: Early life oxytocin treatment improves thermo-sensory reactivity and maternal behavior in neonates lacking the autism-associated gene Magel2
Source: Neuropsychopharmacology. 2022 Apr 8;47(11):1901–12. doi: 10.1038/s41386-022-01313-5 (PMC9485246; doi:10.1038/s41386-022-01313-5)
Supplement: Supplementary file 1 — Supplemental methods and figures [file 41386_2022_1313_MOESM1_ESM.pdf]

SUPPLEMENTARY DATA

Supplemental methods

Corticosterone immunoassay

P2 mice were separated from their mother and placed on a heating pad for 5 min., then sacrificed and blood samples were quickly collected. Blood serum was separated by centrifugation (5,000 rpm, 20 min) and stored at -80°C. Serum corticosterone concentrations were measured with corticosterone ELISA kit (Enzo Life Sciences, Farmingdale, NY, USA) according to the manufacturer's instructions.

Supplemental figures

Supplemental figure 1.

A;C;E: Before/after graphs illustrating the latency to the first call measured upon exposure at 25°C (red dots) followed by 17°C (blue dots) in WT and in $Magel2^{+/-p}$. (A): At P1 : WT (25°C *vs* 17°C) : 1.56±0.27 ln+1s *vs* 0.31±0.08 ln+1s, n=16; p=0.002. $Magel2^{+/-p}$ (25°C *vs* 17°C) : 1.74 ±0.33 ln+1s *vs* 0.97±0.36 ln+1s, n=13; p=0.19. WT *vs* $Magel2^{+/-p}$ (25°C): 1.56±0.27 ln+1s, n=16 *vs* 1.74±0.33 ln+1s; n=13; p>0.99. WT *vs* $Magel2^{+/-p}$ (17°C): 0.31±0.08 ln+1s, n=16 *vs*0.97±0.36 ln+1s; n=13; p=0.19. (C): At P3: WT (25°C *vs* 17°C) : 2.08±0.32 ln+1s *vs* 1.19±0.27 ln+1s; n=15, p=0.04. $Magel2^{+/-p}$ (25°C *vs* 17°C) : 2.5±0.38 ln+1s *vs* 3.24±0.46 ln+1s, n=16, p=0.07. WT *vs* $Magel2^{+/-p}$ (25°C): 2.08±0.32 ln+1s, n=15 *vs* 2.5±0.38 ln+1s; n=16, p=0.86. WT *vs* $Magel2^{+/-p}$ (17°C): 1.19±0.27 ln+1s, n=15 *vs* 3.24±0.46 ln+1s; n=16, p=0.0005. (E): At P6: WT (25°C *vs* 17°C) : 2.97±0.39 ln+1s *vs* 2.56±0.29 ln+1s, n=12, p=0.71.$Magel2^{+/-p}$ (25°C *vs* 17°C) : 2.33±0.42 ln+1s *vs* 3.90±0.39 ln+1s; n=12, p=0.0027. WT *vs* $Magel2^{+/-p}$ (25°C) : 2.97±0.39 ln+1s, n=12 *vs* 2.33±0.42 ln+1s; n=12, p=0.48. WT *vs* $Magel2^{+/-p}$ (17°C) :

2.56±0.29 ln+1s, n=12 *vs* 3.90±0.39 ln+1s; n=12, p=0.033. Repeated-measures (Temperature) Two-way ANOVA, Bonferroni's post-test.

B;D;F: Bar graphs comparing animals responsive rate of coolness-stimulated USV between WT and Magel2$^{+/-p}$ neonates from P1 to P6. At P1, WT: 87.5±8.5%, n=16 *vs* Magel2$^{+/-p}$: 58.82±12.3%; n=17; p<0.0001 (B). At P3, WT: 73.33±11.82%; n=15 *vs* Magel2$^{+/-p}$: 20±10.69%, n=15; p<0.0001 (D). At P6, WT: 50±15.08%; n=12 *vs* Magel2$^{+/-p}$: 7.69±7.69%; n=13; p<0.0001 (F). Fisher's exact test.

G-H: Total number of calls over the age in WT (black lines) and *Magel2*$^{+/-p}$ (orange lines) upon ambient (25°C) (G) and cool exposure (17°C) (H). At P2,17°C WT : 200.1±29.85; n=16 *vs* *Magel2*$^{+/-p}$*:* 105.2±23.7; n=17; p=0.0238. At P3, 17°C: WT: 201.6±35.31; n=16 *vs Magel2*$^{+/-p}$: 48.24±10.49; n=17; p<0.0001. Repeated-measures (age) Two-way ANOVA, Bonferroni's post-test.

Data are presented as mean±SEM.

Supplemental figure 2.

A: Experimental procedure for corticosterone assay. After room habituation, neonates are separated from the dam, placed on a heating pad for 5 minutes and blood samples are collected just before the USV recording.

B: Corticosterone plasma levels in female and male of WT and *Magel2*$^{+/-p}$. Males: WT: 12,974±6,711 ng/ml; n=6 *vs Magel2*$^{+/-p}$: 24,328±1,971 ng/ml; n=7, p=0.4740. Females: WT 19,983±4,425 ng/ml; n=7 *vs Magel2*$^{+/-p}$: 28,831±10,314 ng/ml; n=6, p=0.6723. Comparisons between conditions revealed no significant difference. Two-way ANOVA, Bonferroni's post-test.

Data are presented as mean±SEM.

Supplemental Figure 3. Coolness reactivity in WT upon repeated or reverse temperature exposures

A-C: Before/after graphs illustrating the latency to the first call measured upon a repeated exposure at 25°C (red dots) in new independent WT-only cohorts at P2 (**B**) and P3 (**C**). Wilcoxon test.

D-F: Before/after graphs illustrating the latency to the first call measured upon exposure at 17°C (blue dots) followed by 25°C (red dots) in new independent WT-only cohorts at P2 (**E**) and P3 (**F**). P2 WT (17°C *vs* 25°C): 0.61±0.0.18 ln+1 s *vs* 2.03±0.43 ln+1 s; n=14, p=0.0.0052; P3 WT: 0.25±0.18 ln+1 s *vs* 1.40±1.05 ln+1 s; n=8, p=0.039. Wilcoxon test.

G-H: Bar graphs representing responsive rate of coolness-stimulated USV at P2 (**G**) and P3 (**H**). P2 WT: 25°C-17°C: 73.33±11.82 %, n=15; 25°C-25°C: 41.18±12.30 %, n=17; 17°C-25°C: 85.71±9.70 %, n=14. P3 WT: 25°C-17°C: 73.33±11.82 %, n=15; 25°C-25°C: 25.00±16.37 %, n=8; 17°C-25°C: 66.67±16.67 %, n=9. Fisher's exact test.

Data are presented as mean±SEM.

Supplemental figure 4.

A;C: Before/after graphs represent the latency to the first call measured at 25°C (red squares) and 17°C (blue squares) in Magel2$^{+/-p}$ treated with OT (A): 2.03± 0.39 ln+1s vs 0.59±0.25 ln+1s; n=13; p=0.0049), and AVP (C): 1.44±0.39 ln+1s *vs* 0.28±0.16 ln+1s; n=8; p=0.0156. Wilcoxon test.

B;D: Bar graphs showing animals responsive rate of coolness-stimulated USV in Magel2$^{+/-p}$ untreated or treated with OT (B): 40±16.33 %; n=9 *vs* 76.92±12.16 %; n=13; p<0,0001), or AVP (D): 75±16.37 %; n=8; p<0.0001. Fisher’s exact test.

E: Latency to the first call measured at 25°C (red dots) and 17°C (blue dots) in vehicle (Veh) or TGOT-treated WT. Veh (25°C vs 17°C) : 1.48±0.26 ln+1s *vs* 0.55±0.13 ln+1s, n=15; p=0.0065. TGOT (25°C vs 17°C) : 1.71± 0.37 ln+1s *vs* 0.16± 0.05 ln+1s, n=11; p=0.0002. Veh vs TGOT (25°C): 1.48±0.26 ln+1s, n= 15 *vs* 1.71± 0.37 ln+1s, n=11; p>0.99. Veh vs TGOT (17°C): 0.55±0.13 ln+1s, n= 15 *vs* 0.16± 0.05 ln+1s, n=11; p=0.39. Repeated-measures (temperature) Two-way ANOVA, Bonferroni’s post-test.

F: Responsive rate of coolness stimulated USV in untreated WT compared with TGOT-treated WT: 73.33±11.82 %; n=15 *vs* 81.82±12.20 %; n=11; p=0.2393. Fisher's exact test.

G: Total number of calls recorded during 5 minutes in Magel2$^{+/-p}$ treated with vehicle (41.3±16.48, n=10) and compared with OT (97.31±18.16%, n=13, p=0.45), or TGOT (198.50±23.45 %, n=13, p=0.006), or AVP (205.30±44.03 %, n=10, p=0.0035). Kruskal-Wallis test, Dunn's post-test.

H: Total number of calls in untreated WT compared with TGOT-treated WT at 17°C: 200.1±29.85; n=16 *vs* 255.5±39.92; n=11; p=0.5039. Mann Whitney test.

Data are presented as mean±SEM

Supplemental figure 5. Extracellular signal-regulated kinase (ERK) signaling after cool exposure and oxytocin treatment

A-B: Representative Western blots of cerebral Erk and phosphorylated Erk (P-Erk) issued from WT (A) and *Magel2*$^{+/-p}$ (B) treated with vehicle (Veh) and exposed to 25 or 17°C.

C-D: Western-blots quantification: WT+Veh (25°C): 0.47±0.07 *vs* WT+Veh (17°C): 0.16±0.03; n=6; p=0.0022 (C). Magel2$^{+/-p}$+Veh (25°C): 0.56±0.03; n=7 *vs* Magel2$^{+/-p}$+Veh (17°C): 0.58±0.07; n=5; p=0.7424 (D); Mann Whitney test.

E-F: Representative Western blots of cerebral Erk and phosphorylated Erk (P-Erk) issued from WT (E) and *Magel2*$^{+/-p}$ (F) treated with OT and exposed to 25 or 17°C exposed to 25 or 17°C.

G-H: Western-blots quantification: WT+OT (25°C): 1.32±0.11, n=5 *vs* WT+OT (17°C): 0.76±0.1; n=4; p=0.0317 (G). Magel2$^{+/-p}$+OT (25°C): 1.08±0.09; n=6 *vs* Magel2$^{+/-p}$+OT (17°C): 0.62±0.12; n=6; p=0.0087 (H); Mann Whitney tests.

Data are presented as mean±SEM.

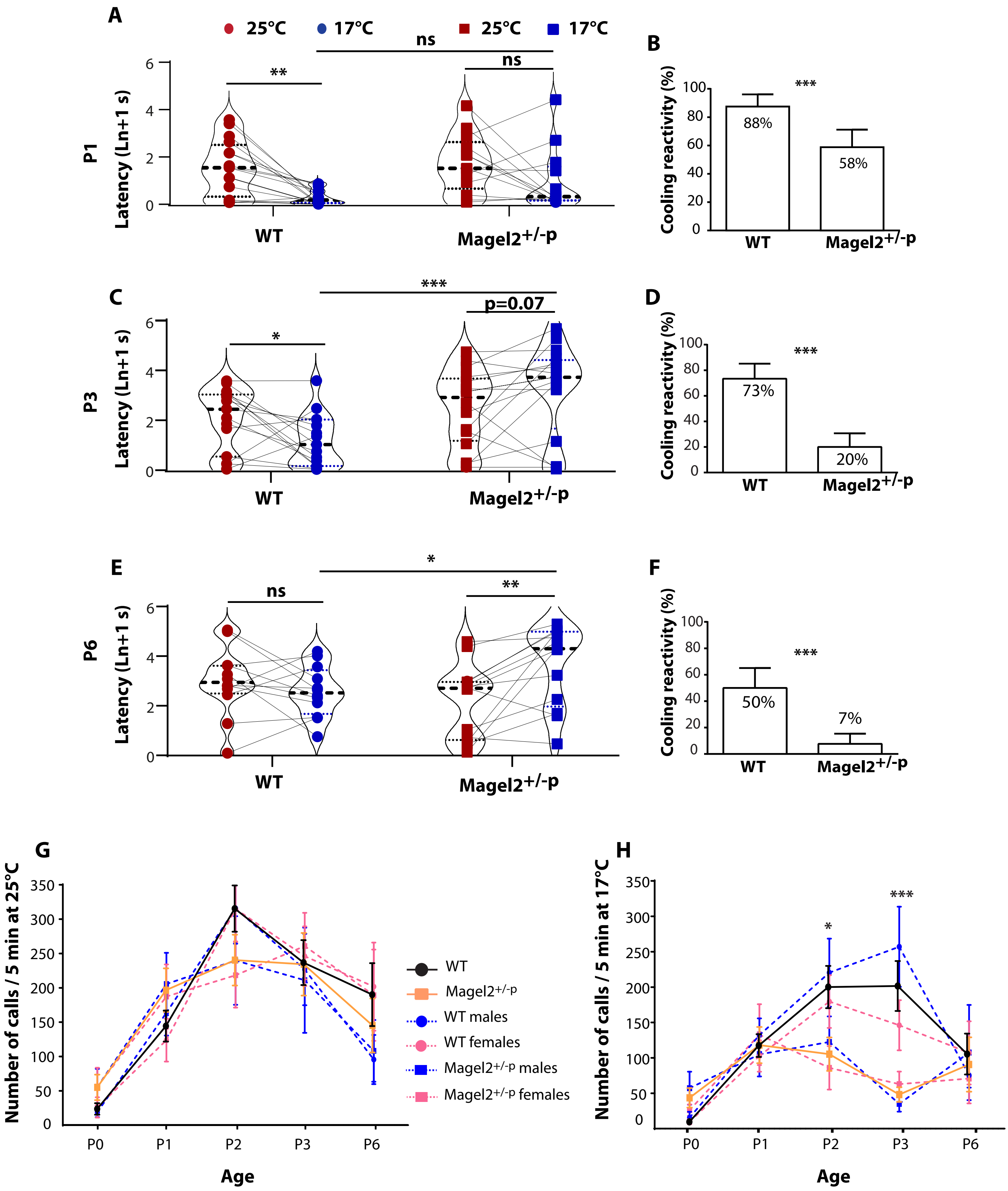

Supplemental Figure 1

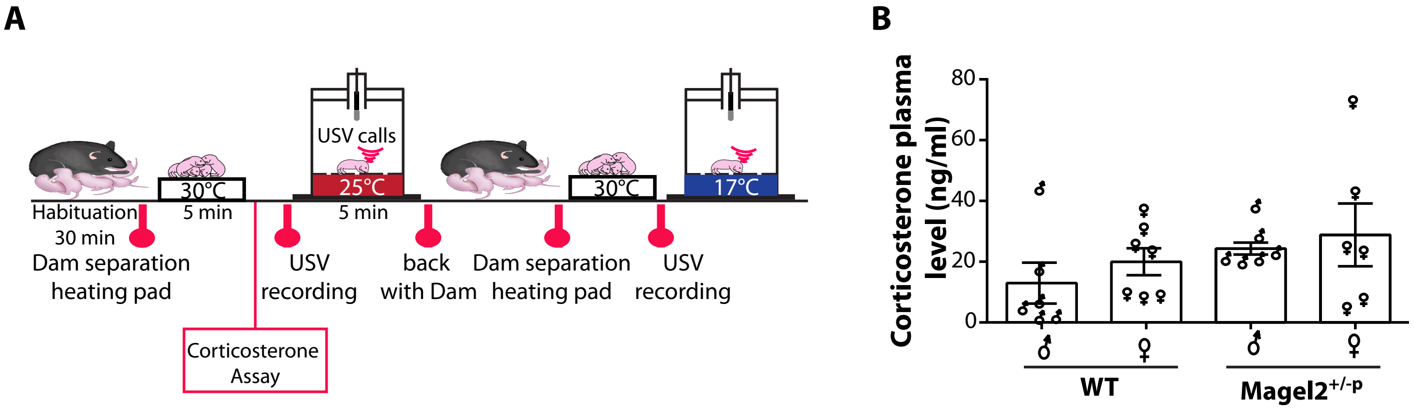

Supplemental Figure 2

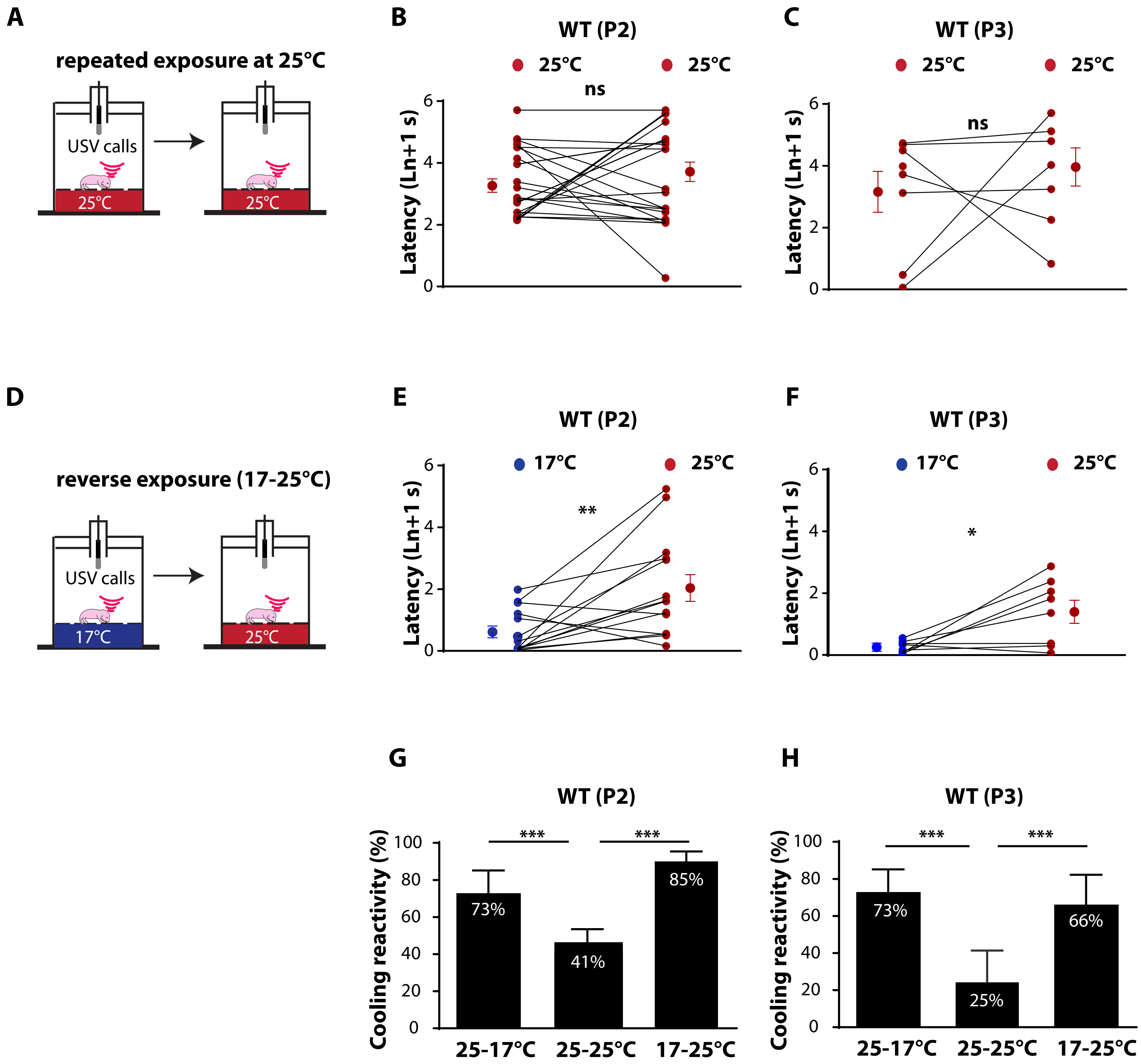

Supplemental Figure 3

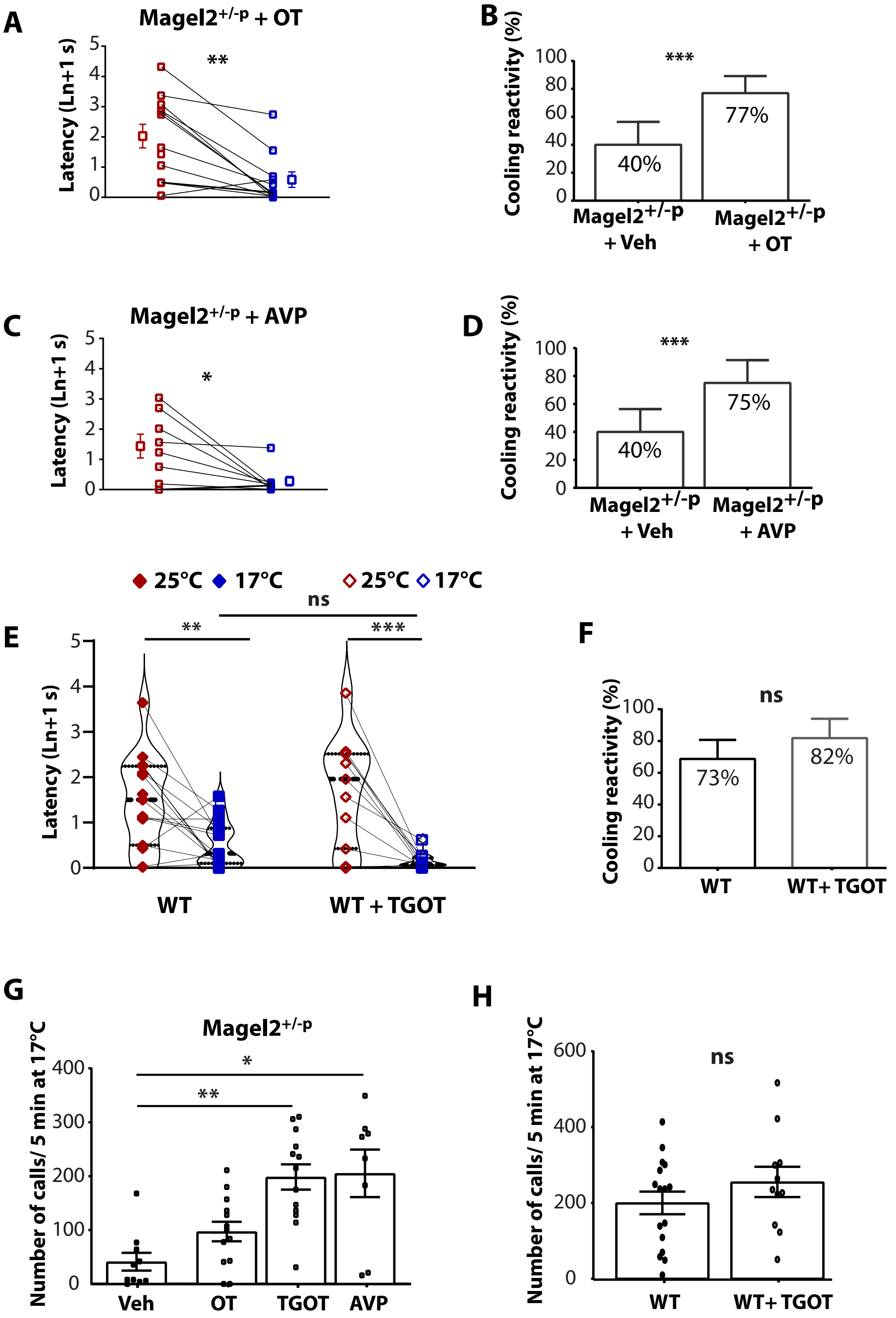

Supplemental Figure 4

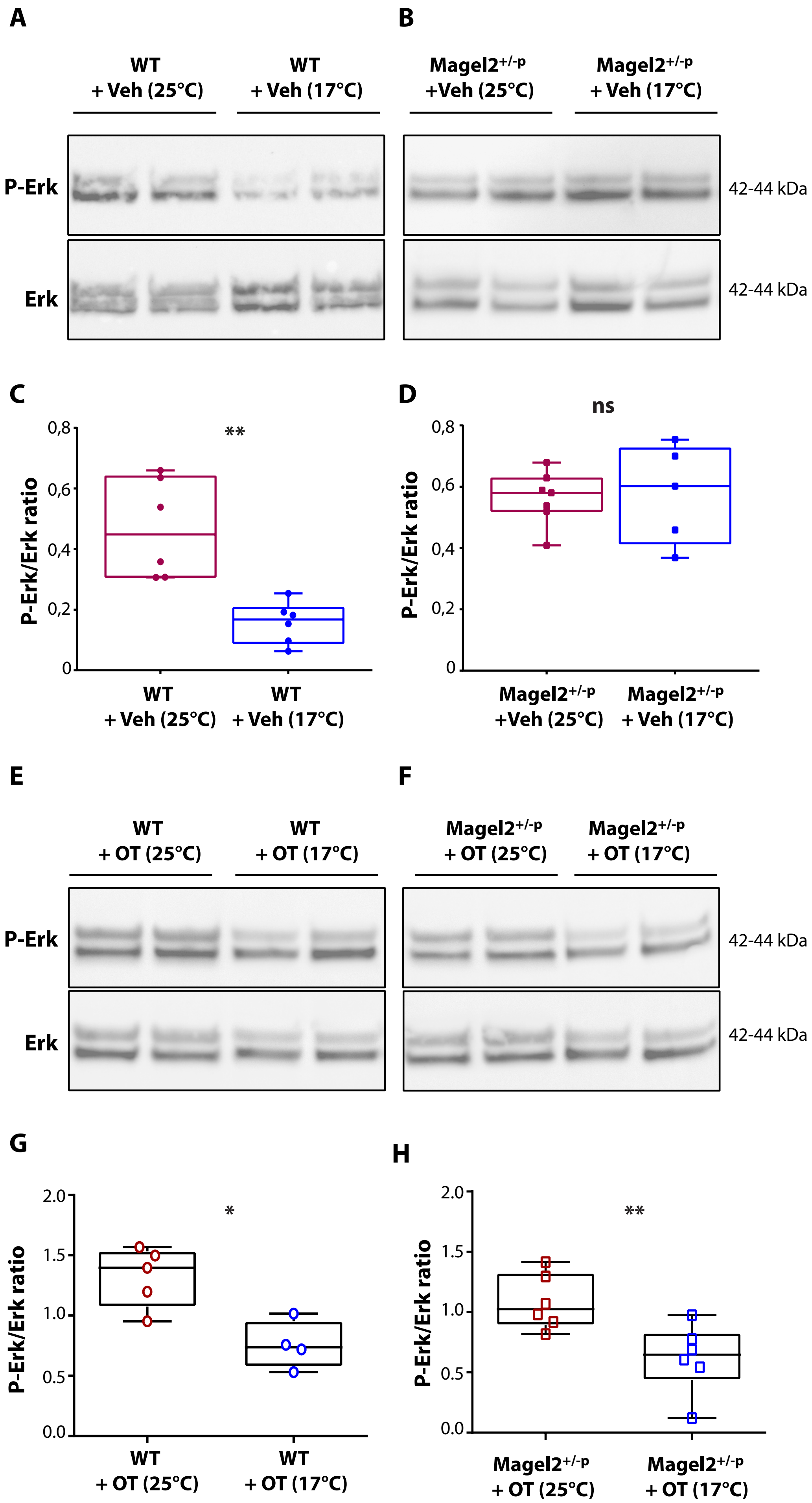

Supplemental Figure 5
